# A Morphological Study of HLA-DR-Immunopositive Cells in Multiple Sclerosis Lesions and Their Implications for Pathogenesis

**DOI:** 10.3390/diagnostics14192240

**Published:** 2024-10-08

**Authors:** Murad Alturkustani, Lee-Cyn Ang

**Affiliations:** 1 Department of Pathology, Faculty of Medicine, King Abdulaziz University, Jeddah 21589, Saudi Arabia; 2 Department of Pathology and Laboratory Medicine, Western University, London, ON N6A 5C1, Canada; 3 Pathology and Laboratory Medicine, London Health Sciences Centre (LHSC), London, ON N6A 5W9, Canada

**Keywords:** multiple sclerosis, HLA-DR-immunopositive cells, macrophages, ramified microglia, chronic active plaques

## Abstract

Background: Multiple sclerosis (MS) is characterized by white matter demyelinating plaques, which can be classified as active, chronic active, or chronic inactive based on the extent of demyelination, cellularity, and inflammation. Microglia and macrophages play a central role in these processes. This study aimed to investigate the morphological characteristics of HLA-DR-immunopositive cells in these plaques to improve our understanding of the roles of these cells in MS plaques. Methods: This study is a retrospective post-mortem histopathological study. We analyzed 90 plaques from 6 MS cases. Of the plaques studied, 77 were grouped into three categories: 28 active, 34 chronic active, and 15 chronic inactive. Additionally, five vacuolated white matter lesions, two axonal degeneration lesions, and six lesions with mixed histological features were included. Six control cases were also examined to assess HLA-DR-immunopositive cell expression across various age groups. The cells were classified based on their morphology into two types: round cells without processes (macrophages) and cells with varying processes and shapes (ramified microglia). Results: Both macrophages and ramified microglia were present in all lesion types, with a focus on identifying the predominant cell type. Of the 28 active plaques, macrophages were the primary cell type in 25 plaques, while ramified microglia predominated in 3. In the center of 49 chronic plaques, scattered ramified microglia were observed in 46, with three plaques showing a predominance of macrophages. Among the 34 chronic active lesions, ramified microglia were the main cell type in the periphery of 32 plaques, with the remaining two predominantly exhibiting macrophages. Conclusions: The predominance of macrophages in active lesions and the presence of scattered ramified microglia in the center of chronic plaques are consistent with the phagocytic role of macrophages. Meanwhile, the prevalence of ramified microglia at the periphery of chronic active lesions suggests a potential protective function in maintaining lesion stability.

## 1. Introduction

Multiple sclerosis (MS) is a neurological disorder characterized by demyelinating white matter lesions in the central nervous system (CNS). Microglial and macrophage activities play crucial roles in MS pathogenesis. However, understanding the intricate cellular landscape of these lesions requires further investigation.

Classification of white matter lesions based on pathological features and phagocytic cell activity has long been a focus of MS research [1,2]. One foundational system categorizes lesions as active, chronic active, or chronic inactive based on cellularity and the presence of MHC class II cells [1]. However, the morphology of these cells remains unclear. The role of macrophages in distinguishing between active and inactive plaques is crucial, particularly in chronic MS lesions.

Traditional histopathological studies have focused on the role of macrophages and microglia in MS but often do not distinguish between these two cell types when examining HLA-DR-immunopositive cells. Macrophages and various subtypes of microglia are typically grouped together, which limits insight into their distinct roles within MS lesions [1,2].

Recently, Prineas and Lee, utilizing a comprehensive analysis of different morphological forms of microglia and their subtypes in MS lesions, concluded that macrophages in MS lesions are derived from circulating monocytes rather than from microglia as proposed by traditional studies [3]. In our study, we build upon these findings by applying the comprehensive morphological classification of macrophages and microglial subtypes, focusing specifically on their distribution in the center and periphery of MS lesions. This approach enables us to explore the functional implications of these cells in lesions.

While morphology alone may not definitively reveal cell origin, differentiating HLA-DR-positive cells into round macrophages and ramified microglia has proven valuable in understanding the pathogenesis of adult-onset leukoencephalopathy/leukodystrophy with axonal spheroids (ALAS). The ALAS study showed that the loss of ramified microglia precedes axonal pathology, implying the protective effect of these cells against CNS axonal injury [4].

In this study, we applied the insights gained from our ALAS studies to examine the appearance of distinct populations of HLA-DR-immunopositive cells in the different stages of MS plaque evolution. We hope that our observation of distinct populations of HLA-DR immunopositive cells based on morphology and their locations within the different plaques will provide fresh insights into the pathogenesis of MS white matter lesions.

## 2. Materials and Methods

This study adhered to ethical standards and was approved by the Institutional Research Ethics Committee at Western University (number 105692). Ethical clearance was obtained to conduct research on human tissue samples under the institutional guidelines and principles outlined in the Declaration of Helsinki. The retrospective nature of the study justified the waiver of patient consent, as there was no direct interaction with patients, and strict confidentiality measures were applied to anonymize patient identifiers.

This retrospective study reviewed autopsy records and histopathological slides at the London Health Sciences Centre Pathology Department over a 15-year period. The inclusion criteria were based on a clinical diagnosis of multiple sclerosis (MS), confirmation in the autopsy report, and the availability of routine histopathological and immunohistochemical slides. The initial cohort included 12 MS cases; however, two cases were excluded due to acute MS variants (Balo’s concentric sclerosis and Marburg forms), and four cases were excluded because detailed clinical histories were unavailable and the duration of MS was unknown. The final cohort of six MS cases fulfilled all the inclusion criteria and were included in the study. Both authors are experienced neuropathologists, and both reviewed and analyzed the samples.

For control cases, six non-MS autopsies were selected to investigate HLA-DR-immunopositive cell morphology within MS lesions. The control cases were selected to provide a reference for HLA-DR immunostaining across different age groups, including individuals aged 9–57 years without diagnosed neurological disorders. The causes of death in these cases varied: one case involved drowning, three cases involved sudden unexpected death in epilepsy (SUDEP), one case involved sudden death with no identifiable cause or brain pathology, and one case involved necrotizing fasciitis with no significant pathological alterations in the brain. All control cases displayed no significant neuropathological findings (as anticipated), rendering them suitable control subjects. The HLA-DR scores across all control cases were low, with a slight tendency to increase with age. However, all cases consistently scored 1, reflecting a stable and minimal level of microglial activation. The inclusion of these control cases aimed to demonstrate the spectrum of neuroinflammation observed in aging and non-MS conditions, serving as a baseline for comparison with the MS cases.

Six cases were clinically diagnosed as MS and confirmed in the autopsy report. Tissue samples were obtained from patients who underwent post-mortem autopsies. Brain specimens were fixed in 10% buffered formalin. Coronal sections of the cerebral hemispheres, sagittal or parasagittal sections of the cerebellum, and horizontal sections of the brainstem were obtained. Representative sections from each case were subjected to microscopic examination to ensure comprehensive coverage of the brain. The spectrum of white matter lesions included active, chronic active, and chronic inactive demyelinating lesions, axonal degeneration, and unclassified white matter lesions, as they did not fit any of the previous categories.

All available plaques from each case were evaluated. We conducted a comprehensive examination of 90 white matter lesions obtained from the selected cases. Lesions were classified into distinct categories: demyelinating lesions when myelin loss exceeded axonal loss, axonal degeneration when myelin loss was proportional to axonal loss, and vacuolated white matter when the preserved myelinated area displayed vacuolation within the white matter but with preserved myelin structure.

All lesions were examined using the following stains and immunostains: Luxol Fast Blue–Hematoxylin and Eosin (LFB-HE) for the visualization of myelin and tissue structure; mouse monoclonal anti-HLA-DR antibody (1:200; clone CR3/43; Dako, Carpinteria, CA, USA) to label HLA-DR-immunopositive cells; Amyloid Precursor Protein (APP; Chemicon, Temecula, CA, USA) to detect early axonal injury; Glial Fibrillary Acidic Protein (GFAP), (Dako, Carpinteria, CA, USA) to identify astrocytic gliosis; and Neurofilament Protein (NFP), (SMI31; Covance, Berkeley, CA, USA) to assess axonal density and integrity. All slides underwent automated immunohistochemistry utilizing the Dako Autostainer Link 48, with visualization conducted using the Dako Envision Flex kit detection system. All LFB-HE stains were performed as part of the routine autopsy workup. Additional HLA-DR immunostaining was occasionally required.

Well-myelinated white matter or normal-appearing white matter (NAWM) was classified as those with no pathological alterations by HE-LFB staining and other immunostains (used as internal control tissue) and those with pathological alterations only apparent by immunostaining, especially those with increased expression of HLA-DR immunopositive cells.

**Figure 1:**
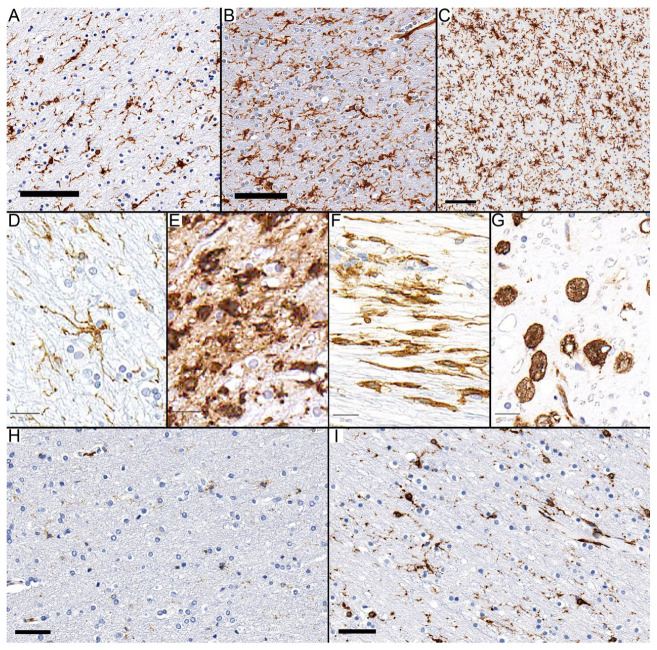
Definitions and Scoring of HLA-DR Immunopositive Cells. (**A**–**C**) Illustrations of HLA-DR scoring: (**A**) score 1, (**B**) score 2, and (**C**) score 3. (**D**–**G**) Classification of HLA-DR immunopositive cell types (**D**) reactive microglia, (**E**) activated microglia, (**F**) “wall-type” microglia (ramified microglia includes (**D**–**F**)), and (**G**) (macrophages). (**H**,**I**) The lowest (**H**,**I**) highest density of HLA-DR immunopositive cells in control cases (both considered score 1). Scale bar: (**A**–**C**): 100 µm; (**D**–**G**): 20 µm; (**H**,**I**): 50 µm.

All slides were scanned using an Aperio scanner, and images were viewed with Aperio ImageScope software (v12.4.3.5008). Digital images were captured for analysis. We scored HLA-DR immunostaining within the lesions semi-quantitatively based on the visual estimation of the proportion of immunopositive cells occupying the lesion (Figure 1A–C). The scoring system was as follows: score 1 (less than 25% of immunopositive cells within the lesion); score 2 (between 25% and 75% of immunopositive cells within the lesion); and score 3 (more than 75% of immunopositive cells within the lesion). The morphology of HLA-DR-immunopositive cells was categorized into two primary phenotypes: round/macrophage (characterized by cells with no apparent processes) and ramified microglia (characterized by cells with discernible processes) (Figure 1D–G). These ramified microglia were further subclassified as “reactive” (thin and elongated processes) and “activated” (thicker and shorter processes) with the subtype “wall type”, which refers to HLA-DR immunopositive spindle cells with elongated processes [3]. However, distinguishing between these subtypes was challenging in some cases; thus, all three were grouped under the “ramified” category.

In analyzing lesions, we examined both the center and periphery, as well as the adjacent white matter, employing the following criteria: active lesions were defined by an HLA-DR score of 2 or more in the center of the lesion, regardless of the score in the peripheral or adjacent white matter, and chronic lesions were characterized by HLA-DR scores of 1 in the center of the lesion. The latter was subdivided into active and inactive according to the HLA-DR immunopositive cell score in the periphery of the lesion. The designation “active” was considered if the density of HLA-DR immunopositive cells was 2 or 3 at the periphery of the lesion.

## 3. Results

In control cases, microglia predominantly exhibited a ramified morphology characterized by thin processes, with no significant presence of the round/macrophage phenotype. Notably, while there was an increase in HLA-DR immunopositivity with advancing age, all control cases were scored as 1, signifying a relatively low level of immunopositive cells, despite the apparent age-related trend (Figure 1H,I).

**Figure 2:**
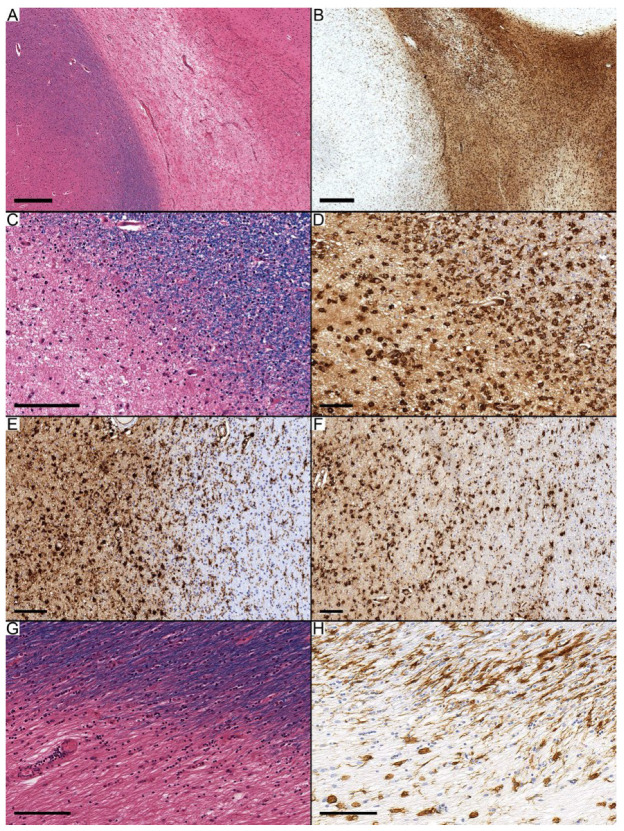
Active lesions with different forms of HLA-DR immunopositive cells. (**A**–**F**) Observations from Case 3. (**A**,**B**) Low magnification images showing active demyelination. (**C**,**D**) Macrophages scored 2 in the center, with activated microglia scoring 3 at the periphery. (**E**) Activated microglia scored 3 in the center and 2 at the periphery. (**F**) Activated microglia scored 2 in the center and 1 at the periphery. (**G**,**H**) The active lesion in Case 5 showed macrophages scored 2 in the center, with “wall-type” microglia scoring 2 at the periphery. Scale bars: (**A**): 400 µm; (**B**): 500 µm; (**C**–**G**): 100 µm.

Table 1 and Table S1 summarize the clinical and pathological findings in the six MS cases. There were 77 MS plaques in total, and these were classified into three main categories: 28 active, 34 chronic active, and 15 chronic inactive. The active lesions (Figure 2A–F) were characterized by increased cellular components composed of inflammatory cells (lymphocytes and HLA-DR immunopositive cells) and astrocytes; the latter typically were reactive with large eosinophilic cytoplasm (i.e., gemistocytes). The macrophages contain myelin degradation products. Among the 28 active lesions, macrophages predominantly occupied the center of 25 lesions, while activated microglia were observed in the remaining three (two with a score of 2, one with a score of 3). Macrophages scored 2 in 16 lesions and scored 3 in 9 lesions. The periphery of these lesions varied; only three contained macrophages, while 25 featured various types of ramified microglia, with the most common subtype being activated microglia (score 2) in 14 out of 25 cases. One of the active lesions in Case 5 showed macrophages with a score of 2 in the center, and “wall-type” microglia scored 2 at the periphery (Figure 2G,H).

**Figure 3:**
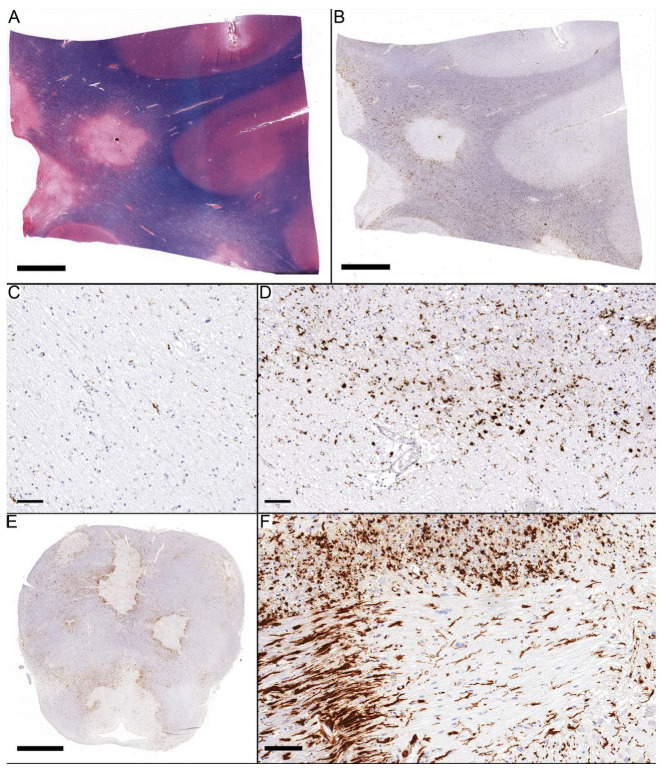
Chronic Active Lesions. (**A**,**B**) Four demyelinated foci with varying activities at their peripheries. (**C**) The center of chronic lesions is characterized by scattered reactive microglia. (**D**) Macrophage scored 1 in the center, with activated microglia scoring 2 at the periphery. (**E**,**F**) Demyelinated foci in the Pons show a prominent edge. (**F**) Microglia morphology at the edge displays “wall-type” in transverse fibers and activated microglia in descending corticospinal tract fibers. Scale bars: (**A**,**B**): 4 mm; (**C**,**D**,**F**): 100 µm; (**E**): 3 mm.

Chronic lesions were characterized by scarce cellularity in the center, including few inflammatory cells (Figure 3A–C) and few recognizable astrocytes. However, GFAP highlighted diffuse fibrillary processes in the background with few gemistocytes, as seen in active lesions. The distinction between active and inactive cells depends on the presence of inflammatory cells at the lesion border. To be considered an active border, the degree of inflammatory cells should have a score of 2 or more (Figure 3D–F).

The center of chronic active lesions contained different types of ramified microglia in 33 of 34 lesions, with the reactive subtype being the most prevalent in 14 lesions. Only one lesion showed a predominance of macrophages. The periphery mostly contained ramified microglia in 32 lesions (31 were the activated type), while macrophages were predominant in only two lesions.

**Figure 4:**
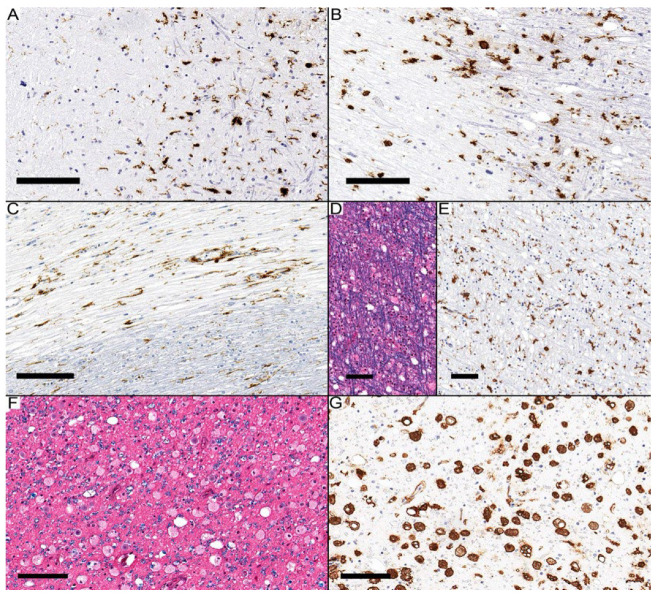
(**A**) Reactive microglia scored 1 in the center, and activated microglia scored 1 at the periphery. (**B**) Macrophages scored 1 in the center, and reactive microglia scored 1 at the periphery. (**C**) Activated microglia scored 1 in the center, with “wall-type” microglia scoring 1 at the periphery from Case 5. (**D**,**E**) Case 4 presents with vacuolated white matter. (**F**,**G**) An example of axonal degeneration is shown in Case 1. Scale bars: (**A**–**C**,**F**,**G**): 100 µm; (**D**,**E**): 50 µm.

Of the 15 chronic inactive lesions, 13 contained various types of ramified microglia at the center, with the activated subtype being the most common in 9 lesions. Macrophages were predominant in the two lesions. The periphery contained ramified microglia exclusively, including eight reactive, six activated, and one “wall” type (Figure 4A–C).

Other white matter lesions were not typically demyelinating, like vacuolated white matter, which showed prominent vacuoles but no definite demyelinated axons (Figure 4D,E). There were examples of axonal degeneration where axonal loss was similar to myelin loss, and most of the HLA-DR immunopositive cells were macrophages (Figure 4F,G). The six difficult-to-classify white matter lesions were as follows: One lesion in Case 1 exhibited prominent axonal degeneration, accompanied by activated ramified microglia with a score of 2 in the center and 1 at the periphery, a pattern not typically associated with axonal degeneration. In Case 4, three lesions were noted: two presented a mixture of axonal degeneration and demyelination, while one showed well-myelinated areas but with a score of 3 for activated ramified microglia. This pattern resembles what is described as a pre-active microglial collection but covers a broader area. In Case 5, the final two lesions primarily consisted of demyelinating foci, with reactive ramified microglia scoring 2 in the center and 1 at the periphery, which did not fit within any of the three demyelinating categories.

## 4. Discussion

Our study presents an analysis of HLA-DR-immunopositive cell morphology in MS lesions and in control cases. The morphology of the predominant HLA-DR immunopositive cells in MS lesions is variable. It tends to correlate with the activity of the lesions (active vs. chronic) and the site (peripheral or central). This variability may reflect the pathogenesis of the lesions and link the function or effect of these different cells on the lesions.

The correlation between the morphology and function of HLA-DR-immunopositive cells is essential for our interpretation of the results. A review of the relationship between microglial morphology and function concluded that although morphology does not directly reflect function, it can be helpful for inferring functional characteristics [5,6]. Typically, ramified microglia with thin processes are associated with surveillance functions [7], whereas the amoeboid phenotype (macrophages) is linked to phagocytic activity [5,6]. Intermediate forms, featuring fewer processes and larger cell bodies, present a more complex challenge in predicting function, although they appear to possess phagocytic capabilities [5,6]. Several variables complicate the interpretation of microglial roles in MS lesions [8], limiting definitive conclusions regarding the significance of various microglial morphologies. Nonetheless, the observed differences suggest a potential correlation and underscore the need for further research in this area. The interpretations suggested in this preliminary study could further our understanding of microglial morphology and its roles in MS.

In the center of the most active lesions (25/28), the most demyelinated areas were marked by macrophages engulfing myelin degradation products at different stages of activity. This aligns with the known phagocytic function of macrophages and confirms their primary role in ongoing demyelination. The visibility of ramified microglia in these areas is limited. However, the origin of these macrophages remains controversial. While early studies suggested that they originate from microglia, Prineas and Lee’s examination of 25 archival MS tissue samples concluded that myelin phagocytes mainly originate from circulating monocytes [3].

The centers of most chronic lesions (44/49) predominantly feature various types of ramified microglia, all scoring 1 but in lower quantities than in normal-appearing white matter (NAWM). The presence of ramified microglia at the center of chronic lesions indicates that these cells do not completely disappear after macrophages dominate the active lesions. However, it is unclear whether these microglia are remnants of previous microglia or represent new cells that have appeared in these areas. The shift in cell type from active to chronic lesions indicates that macrophages are gradually cleared from the centers of the MS lesions.

However, the dense ramified microglia phenotype at the edge of both active and chronic active lesions could be interpreted differently. Possible interpretations of these cells include (1) a nidus for the expansion of demyelinating lesions (the most accepted in the literature), (2) a protective role against the expansion of demyelinating lesions, or (3) a combination of different roles that require in-depth investigation.

The first possibility is widely accepted in the literature, as these lesions, also known as mixed active/inactive lesions, are thought to represent ongoing demyelination [9]. They can be further classified according to the presence of myelin degradation products in macrophages and the extent of the hypercellular border [9]. They are mainly observed in MS patients with a progressive course and clinical history of more than 10 years [10]. In the current study, most of the hypercellular edges (28/30) were formed by ramified microglia (mainly in an activated form) rather than by macrophages. Chronic active lesions were most prevalent in Cases 1 and 2, with disease durations of 36 and 45 years, respectively, which is consistent with their known association with longer disease durations in the literature [10].

This possibility does not explain the difference in the morphological forms of HLA-DR immunopositive cells between the center and periphery of the lesion, as both have the same pathological effect (i.e., active demyelination). Although activated ramified microglia can have phagocytic activity [11], they are still considered to be different from the ameboid macrophage phenotype. These observations suggest that the processes occurring in the periphery of the lesions are not the same as those occurring in the center. This opens the door to considering other possible roles of ramified microglia (i.e., protective rather than detrimental functions). It is not known whether the hypercellular rim is present from the beginning or if it appears as a secondary lesion after a long duration. It is difficult to explain their presence by the ongoing demyelination for such a long duration.

In their review, Pukoli and Vecsei supported the hypothesis that microglia play a critical role in the progression of chronic active lesions, which are also known as smouldering lesions. They described microglia in these lesions as chronically activated, exhibiting a hybrid morphology that combines features of both activated (amoeboid) and ramified forms. This hybrid state is associated with the production of pro-inflammatory cytokines, chemokines, and reactive oxygen species (ROS), which contribute to the persistent inflammatory environment within the lesion [12]. This prolonged activation impedes remyelination and exacerbates axonal damage via mechanisms involving oxidative stress and mitochondrial dysfunction. It has been suggested that these lesions originate from acute inflammatory lesions and undergo continuous low-grade demyelination and neurodegeneration over many years [12].

The second possibility is the protective role against expanding central lesions. This stems from the known protective function of ramified microglia [8]. Ramified microglia are in continuous surveillance mode and rapidly react to injuries [8]. Microglial proliferation is one of the earliest changes in ischemic leukoencephalopathy [13], which suggests that they may act to prevent further damage (i.e., have a protective role). Loss of the ramified microglial reaction may result in expanding white matter lesions, as observed in adult-onset leukoencephalopathy with axonal spheroids [4]. Therefore, we propose the possibility of a protective role for ramified microglia at the cellular edge of demyelinating lesions. This conclusion aligns with the conclusion of Prineas and Lee on the anti-inflammatory role of microglia in MS lesions [3].

This is an alternative to the proposed causative role of ramified microglia reaction in initiating demyelinating lesions from the observation that ramified microglial reaction in the form of microglial nodules is considered the first observable pathology (i.e., precative lesions) in MS lesions [14]. The most accepted interpretation is that it is the precursor lesion that progresses to MS demyelinating lesions and is thus considered detrimental to the pathology. However, given the high frequency of these microglial nodules, Van Horssen et al. concluded that it is unlikely that they all progress to demyelinating lesions [15]. Here, we propose that microglia have a protective response to unknown stimuli that limit the progression of MS lesions in most foci. This is a similar explanation for the role of ramified microglia at the edge of active and chronic active MS lesions.

Other white matter lesions included axonal degeneration, vacuolating white matter, and other lesions that were difficult to classify. The observation of axonal degeneration in some lesions underscores the relevance of axonal pathology in MS [16]. This prompts speculation regarding whether the microglial/macrophage phenotype plays a role in axonal preservation or degeneration. Further research is needed to elucidate the mechanisms underlying axonal damage and their correlation with specific cell populations. Vacuolated white matter lesions are non-specific changes and have been observed in white matter ischemia [13], toxic leukoencephalopathy, autosomal dominant leukodystrophy, and other leukoencephalopathies. Other white matter lesions that did not fall under the classification were difficult to classify and may require further understanding of the different white matter pathologies in MS.

Many reviews on the diverse roles of microglia in MS pathogenesis illustrate the complex relationship between different microglial states and their dual roles in neuroprotection and neurotoxicity, particularly in the context of neuroinflammatory diseases, such as MS, which is characterized by chronic inflammation, demyelination, and neurodegeneration [5,12,17,18,19].

Microglia play a critical role in both the demyelination and remyelination processes. The morphological transition to the “active” amoeboid form is closely associated with phagocytic activity, which is crucial for removing myelin during demyelination. However, this activity is equally important for remyelination, as effective remyelination relies on the appropriate removal of myelin debris. Inadequate or excessive microglial activation can lead to impaired remyelination and progressive neurodegeneration, highlighting the delicate balance required for proper CNS repair mechanisms [17].

The functional states of microglia are complex, ranging from pro-inflammatory to anti-inflammatory, with a spectrum of intermediate states that blend the characteristics of both. Morphological assessments alone may not fully capture the specific roles of microglia in different pathological states, as intermediate forms of activated microglia are associated with various functional roles, including phagocytosis and cytokine production, depending on the specific CNS context. Therefore, it is recommended that both qualitative and quantitative approaches be used to assess microglial morphology. While qualitative assessments typically involve categorizing microglial cells based on their shape, quantitative methods offer deeper insights by measuring specific morphological features, such as cell body size, branch length, and the complexity of branching patterns. These measurements can be correlated with functional markers to better understand the role of microglia in various pathological conditions, including MS [5].

The main limitation of this study is the relatively small sample size and retrospective nature of the study. Potential selection bias in the examined cases. These limitations may have influenced our results and interpretation. Larger and more diverse cohorts are essential to validate our findings and enhance their generalizability.

This investigation presents a novel approach to analyzing the cellular composition of MS lesions by providing a detailed morphological classification of HLA-DR-immunopositive cells. While previous studies have examined cell morphology in MS lesions, they frequently did not differentiate between various subtypes of microglia and macrophages. We implemented a comprehensive morphological classification system to examine the distribution and frequency of macrophages and microglial subtypes across both the center and periphery of active, chronic active, and chronic inactive lesions. This differentiation enables a more nuanced understanding of the cellular dynamics within MS plaques. By quantifying the presence of ramified microglia and macrophages and considering their spatial relationships within lesions, we provide novel insights into their potential roles in lesion progression or containment. To our knowledge, this methodology of analyzing lesion microanatomy with detailed cell type classification has not been previously utilized in this context, underscoring the significance and novelty of our findings.

Understanding the diversity of HLA-DR-immunopositive cells within MS lesions holds promise for clinical application. It may aid in refining diagnostic criteria, predicting the disease course, and guiding targeted therapeutic interventions. Investigating the functional roles of microglia and macrophages in lesion dynamics may reveal novel therapeutic targets for modulating neuroinflammation and neuroprotection in MS. Future research should focus on expanding our understanding of the specific functions and interactions between microglia and macrophages in MS lesions. Longitudinal studies that track phenotypic changes over the course of a disease may provide valuable insights. Investigating the impact of immunomodulatory therapies on microglial/macrophage populations could also uncover novel therapeutic strategies.

In conclusion, our study provides valuable insights into the cellular landscape of MS lesions and controls. The centers of the most active lesions are characterized by the presence of macrophages, underscoring their role in myelin degradation. The peripheries of these lesions are primarily occupied by activated microglia, as opposed to macrophages. It is not yet clear whether activated microglia can be transformed into macrophages. Furthermore, the exact function of activated microglia remains uncertain; however, they may serve a protective role in preventing the expansion of MS lesions, challenging the traditional view that peripheral inflammatory activity is associated with lesion progression. This study introduces alternative perspectives on the roles of these cells, which require further research.

## Figures and Tables

**Table 1:** Clinical and pathological features.

No.	Age	Sex	Duration	White Matter Lesion	No.	HLA-DR Type (Score)	
						Center	Perilesional
1	58	F	36 Y	Active	1	RO (2)	RA (2)
				Chronic active	4	RR (1)	RA (2)
				Axonal degeneration (AD)	2	RO (2)	RR (1)
				AD	1	RA (2)	RA (2)
2	73	M	45 Y	Chronic active	18	RR (1)	RA (2)
				Chronic inactive	3	RR (1)	RA (1)
				Chronic inactive	1	RA (1)	RA (1)
				Vacuolated WM	3	RA (2)	RR (1)
3	50	F	13 Y	Active	3	RO (3)	RA (2)
				Active	1	RO (2)	RO (3)
				Active	2	RO (2)	RA (2)
				Active	2	RO (2)	RA (1)
				Active	1	RO (2)	RO (1)
				Chronic active	1	RR (1)	RA (2)
				Chronic active	1	RA (1)	RO (2)
				Chronic active	1	RA (1)	RA (2)
				Chronic inactive	1	RO (1)	RA (1)
				Chronic inactive	1	RO (1)	RR (1)
				Chronic inactive	1	RR (1)	RA (1)
4	47	F	4 Y	Active	2	RO (3)	RA (2)
				Active	4	RO (2)	RA (1)
				Vacuolated WM	1	RA (2)	RR (2)
				Vacuolated WM	1	RA (1)	RA (1)
				Mixture of AD and demyelination	2	RA (1)	RA (2)
				Normal-appearing white matter	1	RA (3)	RR (1)
5	64	F	10 Y	Active	2	RO (3)	RA (3)
				Active	2	RO (3)	RA (2)
				Active	2	RO (2)	RA (2)
				Active	1	RO (2)	RAS (2)
				Active	1	RO (2)	RO (3)
				Active	1	RA (2)	RA (1)
				Active	1	RA (2)	RA (3)
				Chronic active	1	RO (1)	RA (2)
				Chronic active	1	RR (1)	RO (2)
				Chronic active	1	RR (1)	RAS (2)
				Chronic active	2	RA (1)	RA (2)
				Chronic inactive	6	RA (1)	RR (1)
				Chronic inactive	1	RA (1)	RAS (1)
				Demyelinated focus	2	RR (2)	RR (1)
6	36	M	22 Y	Active	1	RA (3)	RA (2)
				Active	1	RO (2)	RA (2)
				Chronic active	4	RA (1)	RA (2)
				Chronic inactive	1	RA (1)	RR (1)

F: female; M: male; RA: activated microglia; RAS: spindle microglia (“wall” type); RR: reactive microglia; RO: macrophage phenotype; Y: years.

## Data Availability

All relevant data files are available with the corresponding author (MA) upon request.

## Supplementary Materials

The following supporting information can be downloaded at: https://www.mdpi.com/article/10.3390/diagnostics14192240/s1, Table S1: Detailed pathological features of the lesions.
